# Associations Between Subjective Age and History of Incarceration in a Population-Based Sample of Older Adults

**DOI:** 10.1093/geroni/igaf122.936

**Published:** 2025-12-31

**Authors:** Rodlescia Sneed, Sabrina Blank

**Affiliations:** Wayne State University, Detroit, Michigan, United States; Wayne State University, Detroit, Michigan, United States

## Abstract

The number of older adults with history of incarceration (HOI) has increased significantly since the 1990s; however, the psychological effects of incarceration on older adults are understudied. Using pooled data from the 2012 and 2014 waves of the Health and Retirement Study, we examined associations between HOI and older subjective age and explored potential mediators. Participants were considered to have older subjective age if they reported feeling older than their chronological age. We used logistic regression to evaluate the association between HOI and older subjective age, adjusting for demographic characteristics. To examine mediation effects, we performed a Karlson-Holm-Breen (KHB) decomposition analysis using logistic regression. Potential mediators included depressive symptoms, number of chronic conditions, income, number of difficulties with activities of daily living (ADLs), and years of education. We observed a significant association between HOI and older subjective age. (OR: 1.72; 95% CI: 1.44-2.05). Fifty-seven percent of the observed effect of HOI on older subjective age was accounted for by these mediators. Depressive symptoms were the primary mediator, accounting for 72.26% of the mediation effect. Number of chronic conditions, years of education, and number of ADLs accounted for 11.40%, 11.02%, and 7.02% of the mediation effect, respectively. Income did not contribute significantly. Our findings underscore the need to address the mental health needs in this population to promote healthier aging. Further, the significant impact of multiple mediators highlights the interplay of social, physical, and psychological vulnerabilities in this population.

